# Recent Advances in the Modeling of Ionic Liquids Using Artificial Neural Networks

**DOI:** 10.1021/acs.jcim.4c02364

**Published:** 2025-03-27

**Authors:** Adrian Racki, Kamil Paduszyński

**Affiliations:** Department of Physical Chemistry, Faculty of Chemistry, Warsaw University of Technology, Noakowskiego 3, 00-664 Warsaw, Poland

**Keywords:** artificial neural networks, ionic liquids, machine learning, deep learning, QSAR/QSPR, property prediction

## Abstract

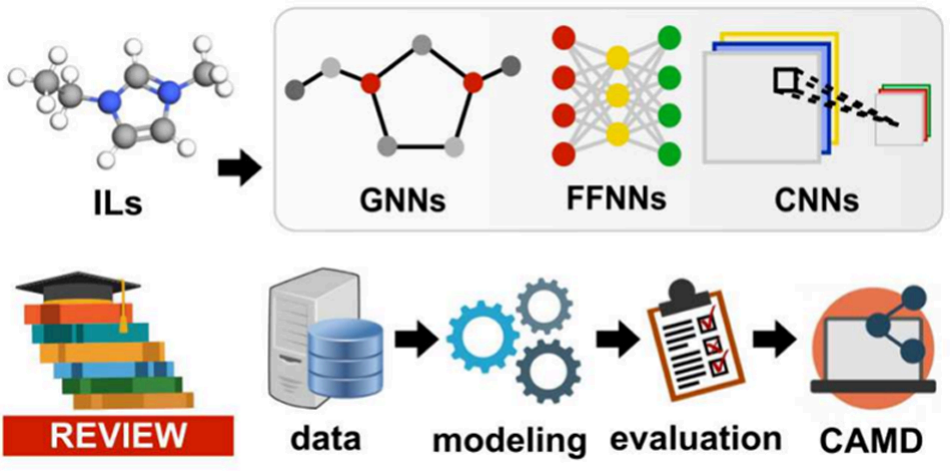

This paper reviews the recent and most impactful advancements in the application of artificial neural networks in modeling the properties of ionic liquids. As salts that are liquid at temperatures below 100 °C, ionic liquids possess unique properties beneficial for various industrial applications such as carbon capture, catalytic solvents, and lubricant additives. The study emphasizes the challenges in selecting appropriate ILs due to the vast variability in their properties, which depend significantly on their cation and anion structures. The review discusses the advantages of using ANNs, including feed-forward, cascade-forward, convolutional, recurrent, and graph neural networks, over traditional machine learning algorithms for predicting the thermodynamic and physical properties of ILs. The paper also highlights the importance of data preparation, including data collection, feature engineering, and data cleaning, in developing accurate predictive models. Additionally, the review covers the interpretability of these models using techniques such as SHapley Additive exPlanations to understand feature importance. The authors conclude by discussing future opportunities and the potential of combining ANNs with other computational methods to design new ILs with targeted properties.

## Introduction

Ionic liquids (ILs) — salts composed of organic cations and organic or inorganic anions that remain liquid at temperatures below 100 °C — possess properties that make them valuable for various applications. These include carbon capture,^1^ solvents for catalytic reactions,^2^ and lubricant additives.^3^ However, selecting the optimal IL for a specific application can be challenging, as their properties depend heavily on the structure of the cation and anion. Balancing key properties such as density, heat capacity, thermal conductivity, and viscosity — particularly for heat transfer fluids — can be difficult, especially when comprehensive and accurate experimental data are lacking for many IL types. Consequently, there is a growing need to develop methods that estimate these properties based on cation/anion structures and well-curated data sets.

Machine learning (ML) offers valuable insights into quantitative structure–property relationships (QSPRs).^4−6^ Significant advancements in this field have resulted in a wealth of literature and various methods tailored to different property prediction problems. Artificial neural networks (ANNs) are particularly prominent and effective among these. That is why this paper focuses on the application of ANNs in this domain.

As noted, several ML approaches have been employed to model the complex relationships inherent in IL-based systems. Some of the most widely used and effective methods include support vector machines (SVM), its least-squares variant (LSSVM), decision trees (DT) and their extended form random forest (RF), as well as boosting algorithms (e.g., XGBoost, LightGBM, CatBoost). Additionally, various ANN architectures have been explored, ranging from simple feed-forward neural networks (FFNN) and convolutional neural networks (CNN) to more advanced models such as graph neural networks (GNN), transformer neural networks (TNN), and deep belief networks (DBN). For an in-depth discussion of these algorithms, readers are referred to works dedicated to this topic.^4,7,8^ Nonetheless, the following sections provide a brief overview of the relevant types of ANNs to establish the necessary context.

Although algorithms not based on neural networks can produce reliable and effective models, they are often surpassed by even single-hidden-layer FFANNs in terms of scalability and generalization to diverse data sets.^9,10^ Given the limitations of non-ANN methods, the authors emphasize the importance of systematizing recent developments in IL property modeling using artificial neural networks. Advances in this field, combined with easy access to substantial computing power, further promote the use of these methods in computer-aided property prediction and molecular design.

ILs present unique challenges for prediction of molecular properties compared to standard molecules due to their inherent ionic composition, complex charge distributions, and long-range electrostatic interactions.^11^ It is also essential to consider inherent data issues in ILs domain, such as size and quality, as they represent critical aspects of property modeling. Accurate evaluation of IL property prediction models differs from standard approaches in the field, as it should account for their ability to generalize to previously unseen cations and anions.^12^ By reviewing recent advancements, we aim to systematically assess the current state of molecular property prediction for ILs relative to state-of-the-art (SOTA) methods, which have increasingly focused on self-learning techniques and language models that leverage vast amounts of unlabeled data to increase the expressivity and generalization capabilities of trained models. Transformer-based models such as Mol-BERT^13^ and SMILES-BERT^14^ pretrain on extensive SMILES data sets, capturing the intricate semantics of molecular structures without relying solely on annotated labels. In parallel, MolCLR^15^ applies contrastive learning to develop robust molecular representations by distinguishing subtle differences in molecular graphs, while XGraphBoost^16^ integrates graph-based approaches with boosting methods to improve predictive performance. Together, these methods exemplify how self-supervised and semisupervised learning can unlock valuable insights from unlabeled data, pushing the boundaries of molecular property prediction. For a broader perspective on these model architectures and performance on various benchmarks, readers are encouraged to explore recent reviews that provide additional insights into this topic.^17−19^

This review aims to highlight the most notable recent efforts (from 2020 onward) in advancing ANN-based IL property prediction. It provides a comprehensive discussion of each stage of machine learning (ML) model development: data preparation, ANN architecture design and optimization, and model evaluation and interpretation. Additionally, section other related work covers noteworthy and innovative studies that do not fit neatly into the other sections but deserve to be mentioned. Finally, last section offers a brief overview and future perspectives on the application of ANNs in IL property prediction. For an analysis of earlier achievements in this field, readers are referred to other reviews.^6,20,21^

## Data Preparation

Data preparation is the most important step in the development of any machine learning model. The workflow primarily involves problem definition, data collection, feature engineering, and data cleaning. Although splitting data into test and training subsets is a necessary step, it is more accurately considered part of the modeling process rather than data preparation, especially in the context of cross-validation, where it is performed iteratively in parallel with model construction.

### Formal Problem Definition

In modern ML applications for predicting IL properties, it is essential to adopt a flexible mathematical framework that accommodates diverse molecular representations, including fixed-length feature vectors, variable-length sequences (e.g., SMILES) and graph-structured data.

Let *D* = {(*x*_*i*_, *y*_*i*_)}_*i* = 1_^*N*^ represent a data set of *N* samples, where *x*_*i*_ denotes the molecular structure of the *i*^th^ IL, and  is the corresponding scalar property value. The goal is to learn a predictive function *f*(·; θ), parametrized by θ, to minimize the loss :
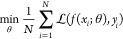


Here, *x*_*i*_ belongs to a feature space , which may be a fixed-length vector , a sequence  (e.g., SMILES), or a graph *x*_*i*_ = (*V*_*i*_, *E*_*i*_) with nodes *V*_*i*_ and edges *E*_*i*_. The predictive function *f* varies by representation: classical regression models use *f*(*x*; θ) = *w*^*T*^*x* + *b*; FNNs apply transformations through layers with activation functions; transformers process sequences via self-attention mechanisms; and GNNs aggregate node and edge information through message-passing mechanisms. The loss function  is typically mean-squared error (MSE) for continuous properties or cross-entropy for classification tasks. Optimization usually relies on gradient-based methods such as SGD or Adam. This framework provides a unified approach for integrating diverse molecular representations and ML models, enabling accurate and adaptable property predictions for ILs.

### Data Collection

The success of any ML-based project depends heavily on the quality and relevance of the data it utilizes. In IL property modeling (as well as in other fields related to physicochemical and/or thermodynamic properties), the accuracy and reliability of the final model are directly tied to the quality of the input *y* data. Fortunately, experimental data on IL properties are relatively easy to obtain, thanks to several public databases, such as ILThermo^22^ and OCHEM.^23^ ILThermo is a web-based database that provides extensive information on over 50 chemical and physical properties of pure ILs, as well as their binary and ternary mixtures with various solvents. OCHEM, on the other hand, is an online platform that not only stores experimental chemical data but also facilitates the creation, training, testing, and sharing of predictive models and data sets.

In some cases, researchers collect their data or use data sets from published studies. This might be necessary if the property of interest is not included in ILThermo or if the researchers choose to focus on a specific subset of the IL domain. The former scenario is common in studies modeling properties such as various types of toxicity^24−28^ or process parameters like reaction yields in CO_2_ cyclization reactions using ILs as catalysts.^29^ The latter occurs when researchers narrow the scope to a specific group of ILs to create a more specialized model.^30,31^ While these specialized models sacrifice generalizability, they often achieve higher precision within their targeted domain.

Not all databases are derived from experimental measurements. For instance, Can et al.^32^ used COSMO*therm* software to calculate the water solubility of ILs. This computational approach enabled the creation of a database containing solubility data for water in 16 137 ILs, which was subsequently used to develop ML models for structure-solubility relationships. The study revealed that anionic descriptors were generally more significant in predicting water solubility, while cationic descriptors had a comparatively smaller impact.

### Feature Engineering

Feature engineering is a critical step in the ML workflow, serving as the bridge between raw data and a model’s ability to learn and make predictions. Raw data often exists in a format that is not suitable for direct use by algorithms. Feature engineering addresses this by transforming and manipulating the data to create a set of features/attributes that are more informative and better suited for the learning process. Selecting the appropriate set of features to accurately describe each IL or chemical compound in the data set is essential, as it directly impacts model performance. Over the years, various methods have been developed to calculate the input features. The most commonly used methods include the group contribution (GC) method, molecular descriptors (MDs) computed through cheminformatics software, basic compound-specific properties (e.g., acentric factor, critical temperature, and pressure), or combinations of these. Models based on these basic compound-specific properties are often used primarily to gain insight into how certain properties depend on the structure and characteristics of the compounds. However, their main limitation is that they cannot predict the properties of ILs or other compounds that do not have experimentally measured data for these specific properties. When modeling temperature- and pressure-dependent properties, it is essential to include temperature and pressure as explicit features in each feature vector. This is particularly important when other features, such as basic compound-specific properties or method-specific descriptors, do not inherently account for temperature or pressure dependence, ensuring that the model captures the influence of these conditions on the target property. Regardless of the feature vector calculation method, selecting statistically significant features is crucial to reduce overfitting, improve model performance, and reduce computational cost by lowering input dimensionality.

**Figure 1:**
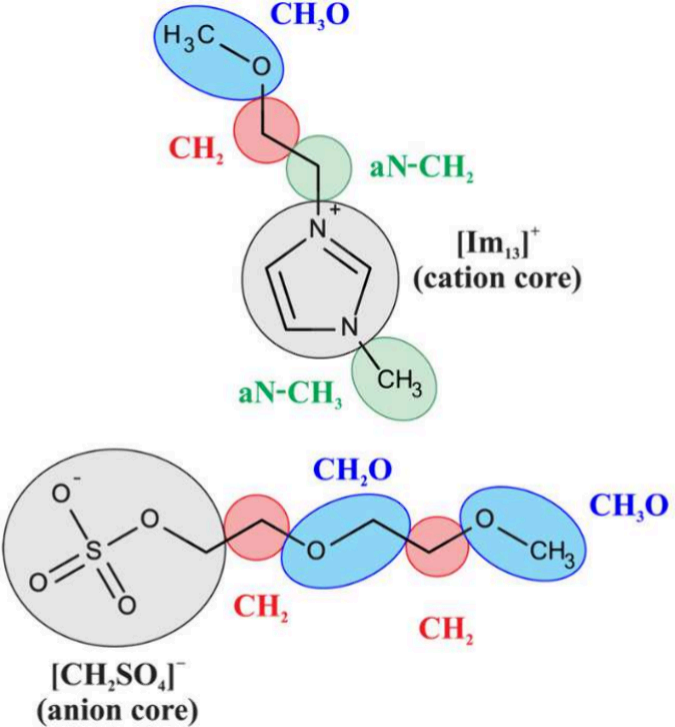
An exemplary group assignment in the GC method. Reprinted with permission from Paduszyński and Domańska.^33^ Copyright 2014 American Chemical Society.

The GC method encodes the chemical structure of cations and anions by counting the occurrences of predefined functional groups.^34^ The selection of these functional groups depends on the specific data set and the expertise of the model developers. Figure 1 illustrates the principles of this method. For more comprehensive coverage of the GC method, readers are referred to relevant literature.^21,35^

MDs offer another way to encode the structure and properties of ions or entire chemical compounds. These descriptors can be computed using free software such as RDKit^36^ and Open Babel,^37^ or commercial tools like CODESSA^38^ and DRAGON.^39^ MDs, which vary in dimensionality, provide a comprehensive representation of chemical structures depending on their “depth”. However, MDs may not be possible to be calculated for all chemical structures, limiting the generalizability of the model. Moreover, applying a trained model to new ions not included in the training/testing phase can be challenging due to the need to compute entirely new MDs for these ions. For instance, descriptors derived from quantum chemical calculations or molecular dynamics simulations require significant computational resources and expertise, making the process of property prediction time-consuming and challenging. Despite these challenges, MDs often help identify the most impactful features during the evaluation phase, offering valuable insights into the model’s predictions.

Evaluating different methods for structural representation is crucial for understanding their effectiveness in property prediction tasks. However, direct comparisons between models built using different methods and data sets can be complicated due to confounding variables. Baskin et al.^40^ conducted a large-scale benchmarking study, comparing machine learning algorithms and encoding methods for six IL properties: density, electrical conductivity, refractive index, surface tension, viscosity, and melting point. They employed four molecular descriptor generation methods: ISIDA-Fr, MolD2, CDK23, and Dragon7. Their results showed that, in most cases, models based on ANNs outperformed other nonlinear algorithms. Additionally, they emphasized that selecting the appropriate representation method is critical for achieving optimal performance with a specific ML model.

Abdullah et al.^41^ conducted a systematic comparison, evaluating FFANNs trained on three different feature sets: structural features learned through GNNs, MDs from RDKit, and a combination of both. Their model, which predicted the ionic conductivity of 2 684 ILs, achieved the best performance when using the combined feature set, with a coefficient of determination (*R*^2^) of 0.937. In comparison, models trained solely on structural and molecular features achieved *R*^2^ values of 0.470 and 0.677, respectively.

### Data Cleaning

Data cleaning is a fundamental step in developing each ML project. Raw data often contain inconsistencies, errors, and missing values, which can severely hinder the learning process of machine learning algorithms, resulting in inaccurate or misleading outcomes. The significance of data cleaning in machine learning is well established.^42^ However, in the rush to advance to model development, this stage is sometimes overlooked or inadequately addressed. Neglecting proper data cleaning can introduce biases, reduce model accuracy, and compromise generalizability. Unfortunately, many of the scientific articles reviewed for this study either do not perform or fail to mention data cleaning practices.

Interestingly, the application of ANNs may offer advantages in scenarios where data preprocessing is minimal. Baran and Kłoskowski,^43^ in their comprehensive study on the use of GNNs for predicting IL surface tension and viscosity, reported improved model performance when using raw, unprocessed data. This suggests that GNNs might effectively handle mislabeled or noisy data by training on diverse IL data sets, leading to smaller generalization errors. Similarly, Feng et al.^44^ investigated the impact of mislabeled data on deep neural network (DNN) performance. They found that even with (40 to 50)% of the data incorrectly labeled, stochastic gradient descent algorithms could distinguish noisy inputs and achieve test errors comparable to models trained on clean data. Their analysis also highlighted the importance of early stopping to prevent overfitting toward noisy data, underscoring a key strategy in mitigating the effects of mislabeled or inconsistent inputs.

### Data Splitting

Splitting data into training and validation sets is critical for reliably evaluating a model’s performance.^45^ The training set is used to fit the model by estimating its unknown parameters, while the validation set is employed to assess the model’s accuracy. This separation is crucial because using the entire data set for training can lead to overfitting, resulting in poor predictions of new data. By reserving a portion of the data set for validation, we can fairly evaluate the model’s performance and address potential overfitting issues before its deployment and applications.

The simplest method for splitting data involves randomly dividing the data set in a specific ratio. However, this single-step random split is rarely sufficient due to potential biases, which may result in misleading performance estimates. To mitigate these issues, data scientists commonly employ cross-validation (CV) or systematic sampling algorithms.^46^ Among these, *K*-fold cross-validation is the most widely used. It partitions the data set into *K* folds, with each fold serving as the validation set once while the remaining folds are used for training. This process is repeated *K* times, and the results are averaged to provide a robust performance estimate. While the CV step is invaluable for model evaluation, it has drawbacks. It can significantly increase computational costs, particularly for large data sets or models with numerous parameters, such as GNNs and CNNs, which are computationally intensive to train. In the context of IL property modeling, CV may yield overly optimiztic results, as it often overlooks the unique influence of individual ions and their combinations on model predictions. Makarov,^12^ in their study on IL melting point prediction, highlighted this limitation and proposed additional cross-validation protocols tailored to ionic liquids: *components out*, *mixtures out*, *anions out* and *cations out*. The *components out* protocol is the most stringent, requiring each ionic pair in the validation fold to include at least one ion absent from the training set. This method closely mimics real-world scenarios, where predictive models must generalize to unseen ion combinations. The *mixtures out* protocol, on the other hand, focuses on validating the model’s ability to predict new ion pair combinations while allowing some overlap (e.g., ILs sharing the same cation but different anions across training and validation sets). The *anions out* protocol excludes all instances of a given anion from the training set, ensuring that only previously unseen anions appear in the validation fold. This approach tests the model’s ability to generalize to novel anionic species while maintaining known cations. The *cations out* protocol applies the same principle to cations, with anions still present in both training and validation sets. Since data sets usually contain more distinct cations than anions, this protocol generally yields lower errors than *anions out*, though differences in chemical diversity and structural complexity may also contribute.

To reduce computational overhead, strategies like the computer-aided design of experiments (CADEX) by Kennard and Stone^47^ and DUPLEX by Snee^48^ were developed to perform a single, representative split into training and validation sets. These methods systematically select representative samples based on data distribution, with the remainder used for validation. A recent enhancement, the SPlit method based on support points, introduced by Joseph et al.,^49^ improves worst-case test performance across various models and can be applied to both categorical and regression data sets. Despite their effectiveness, these systematic methods are seldom used in IL property prediction research.^34^ In our view, they represent an excellent alternative for researchers relying solely on random splits.

In summary, a comparative study by Xu et al.^45^ concluded that no universally optimal data-splitting method exists. The choice should depend on the data set’s size and characteristics, ensuring an appropriate balance between computational efficiency and model performance reliability.

## ML Algorithms and Their Optimization

In this section, we will explore the most commonly used and recently popular ANN architectures in the computational modeling of IL properties. We will also compare the performance of ANNs with other nonlinear supervised ML methods, focusing on their applications, key architectural components, and optimization techniques. However, we will not conduct a standard performance comparison of models from different studies due to several practical challenges. These include the lack of standardized validation protocols, variations in the databases used, and differences in data-splitting methods for training and validation.

Table 1 summarizes selected results from recent work on modeling IL properties using FNNs and CFNNs. It can be seen that, in most cases, ANNs give better results than conventional models, especially when large and diverse data sets are used. However, occasionally, the pursuit of the best possible results leads to the creation of overly complex models that give good results in theory, but their applicability outside the database used is severely limited due to overfitting.

**Table 1:** Summary of Selected Fully Connected Neural Network Models Published Since 2020 for Ionic Liquid Property Prediction

Modeled property	Year	Best model^a^^,^^b^	ILs/data points	Results of the best model (*R*^2^)^c^
SO_2_ solubility (*p, T*)^59^	2020	LSSVM (FFNN)	8/232	0.986
Surface tension of binary mixtures (*T*)^10^	2020	FFNN (ANFIS)	15/1537	0.9998
CO_2_ solubility (*p, T*)^60^	2020	FFNN (SVM)	124/10116	0.9836
Density (*T*)^61^	2020	CFNN (FNN)	15/1248	1.000
Speed of sound (*T*)^61^	2020	CFNN (FNN)	15/1228	1.000
N_2_O solubility (*p, T*)^9^	2021	CFNN (RBFNN)	13/533	0.9985
Ionic conductivity (*T*)^30^	2021	FFNN (SVM)	111/1323	0.991
CO2 solubility (*p, T*)^31^	2021	FFNN	11/430	0.995
Melting point^62^	2022	**FFNN**	1123	0.9
Viscosity of IL/water mixtures (*T*)^63^	2022	FNN	153/8523	0.999
Conductivity (*T*)^64^	2022	**FFNN**	406/4259	0.96
Viscosity of IL/water mixtures (*T*)^65^	2022	CFNN (FFNN)	10/2499	1.000
Ionic conductivity of IL/water mixtures (*T*)^66^	2022	FFNN (MLR)	97/103	0.853
CO_2_ solubility (*p, T*)^53^	2023	FFNN (SVM, LSSVM)	6/546	0.9965
Surface tension of binary mixtures (*T*)^67^	2023	FFNN (SVM, LSSVM)	62/1623	0.9964
Density of binary mixtures (*T*)^68^	2023	FFNN (XGBoost, LightGBM)	129/34754	0.9942
Heat Capacity of binary mixtures (*T*)^68^	2023	FFNN (XGBoost, LightGBM)	17/2504	0.9952
N_2_ solubility (*p, T*)^69^	2023	**FFNN** (SVM)	38/415	0.9886
CO_2_ solubility (*p, T*)^69^	2023	SVM (**FFNN**)	164/13055	0.9763
Electrical conductivity (*p, T*)^70^	2024	FFNN (SVM, XGBoost, RF)	520/7598	0.9977

a Other tested models in the paper in parentheses.

b Acronyms of the models with more parameters than the amount of data have been bolded.

c Coefficient of determination for best model on test data set.

In our view, such comparisons should target a specific parameter and involve independent benchmarking of models from the literature. However, this process is both time-consuming and frequently unfeasible, as many models are not publicly available, rendering them unverifiable.

### Fully Connected Neural Networks

**Figure 2:**
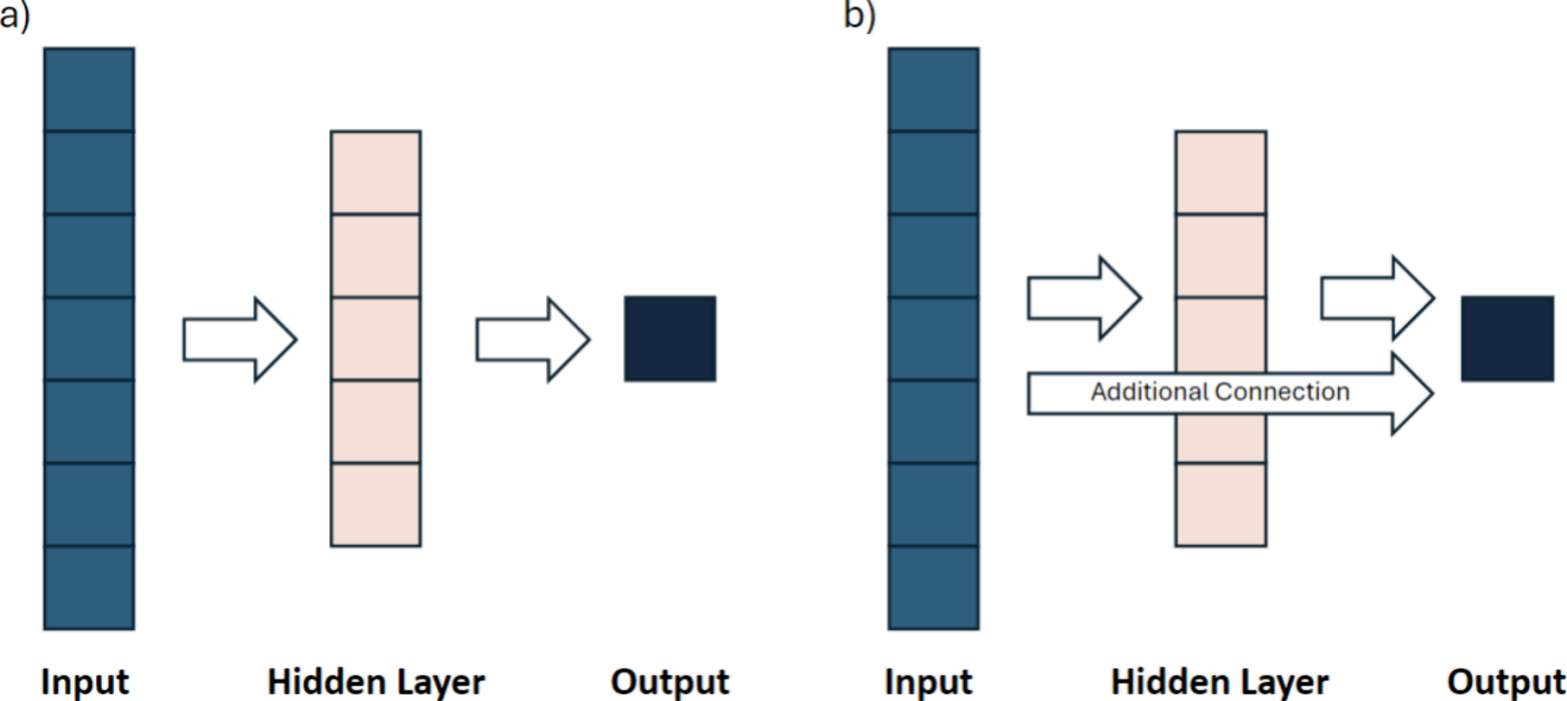
Typical structure of a feed-forward neural network (a) and a cascade-forward neural network (b) with the additional connection between input and output.

The most widely used architecture among ANNs is the simplest one, a fully connected neural network with one hidden layer (often referred to as a multilayer perceptron, MLP), typically in the form of a feed-forward neural network (FFNN) or a cascade-forward neural network (CFNN), as shown in Figure 2. This network passes input data through its layers, with each neuron applying a weighted sum of its inputs followed by a nonlinear activation function. During training, the network adjusts these weights using backpropagation and an optimization function to minimize the error between predicted outputs and actual targets, enabling it to make accurate predictions. In addition to operating individually, this network often serves as a component of more complex architectures, where it functions with learnable weights. Optimization primarily involves selecting the number of neurons in the hidden layer(s), the type of activation function, and the learning algorithm. Input data is typically organized into 2D vectors, where each value corresponds to an input neuron. Specifically, for predicting IL properties, input data may be formatted as sequences of molecular descriptors or counts of building blocks associated with functional groups in ILs.

Recently, researchers have explored various multilayer perceptron variants for predicting IL properties, often comparing them with traditional machine learning methods or different input parameter sets. Shojaeian et al.^10^ examined the impact of input parameters on the efficiency of different ML algorithms in predicting the surface tension of mixtures of ILs and common solvents. They used descriptors such as molecular weight, acentric factor, critical compression factor, critical temperature, critical pressure, and critical volume. To derive mixture properties from pure components, they evaluated both linear and nonlinear approaches based on solvent mole fraction. Their findings showed that an ANN with one hidden layer and 10 neurons outperformed several adaptive neuro-fuzzy inference systems. Additionally, they observed that combining linear and nonlinear rules for calculating mixture MDs could be beneficial for certain features.

Amar et al.^9^ conducted a comparative analysis of the cascade-forward neural network (CFNN), radial basis function neural network (RBFNN), and gene expression programming (GEP) for predicting nitrous oxide solubility in ILs using a data set of 13 distinct salts. CFNNs, similar to feed-forward networks, include connections from the input and each preceding layer to subsequent layers, accommodating nonlinear input-output relationships without ignoring linear connections.^51^ RBFNNs use radial basis functions as activation functions, while GEP, developed by Candida Ferreira in 2001,^52^ mimics biological evolution to generate models. Amar et al. found that CFNN achieved slightly better performance (*R*^2^ = 0.9985) compared to RBFNN and GEP, although the impact of data splitting and cross-validation was not fully explored, limiting the reliability of these results.

Bazooyar et al.^53^ compared FFNN performance (one hidden layer with 8 neurons) to SVM and LSSVM for predicting carbon dioxide solubility in ILs with water impurities. Using a data set of 546 data points for six ILs, they found that the ANN achieved the highest correlation coefficient (*R*^2^ = 0.9965), outperforming SVM (*R*^2^ = 0.9873) and LSSVM (*R*^2^ = 0.9806). However, similar to the work of Amar et al., the influence of data splitting on model performance was not considered.

Single-layer, nonoptimized MLPs often outperform other machine learning methods, but this is not always the case. Lei et al.^54^ compared FFNNs and boosting algorithms (XGBoost and LightGBM) for predicting surface tension and viscosity of IL mixtures. On a data set of 3451 data points, XGBoost slightly outperformed ANN for surface tension prediction (*R*^2^ of 0.9829 vs 0.9681). For viscosity prediction, ANN outperformed XGBoost on a larger data set of 28 435 points (*R*^2^ of 0.9582 vs 0.8597) but required significantly longer computation times.

**Figure 3:**
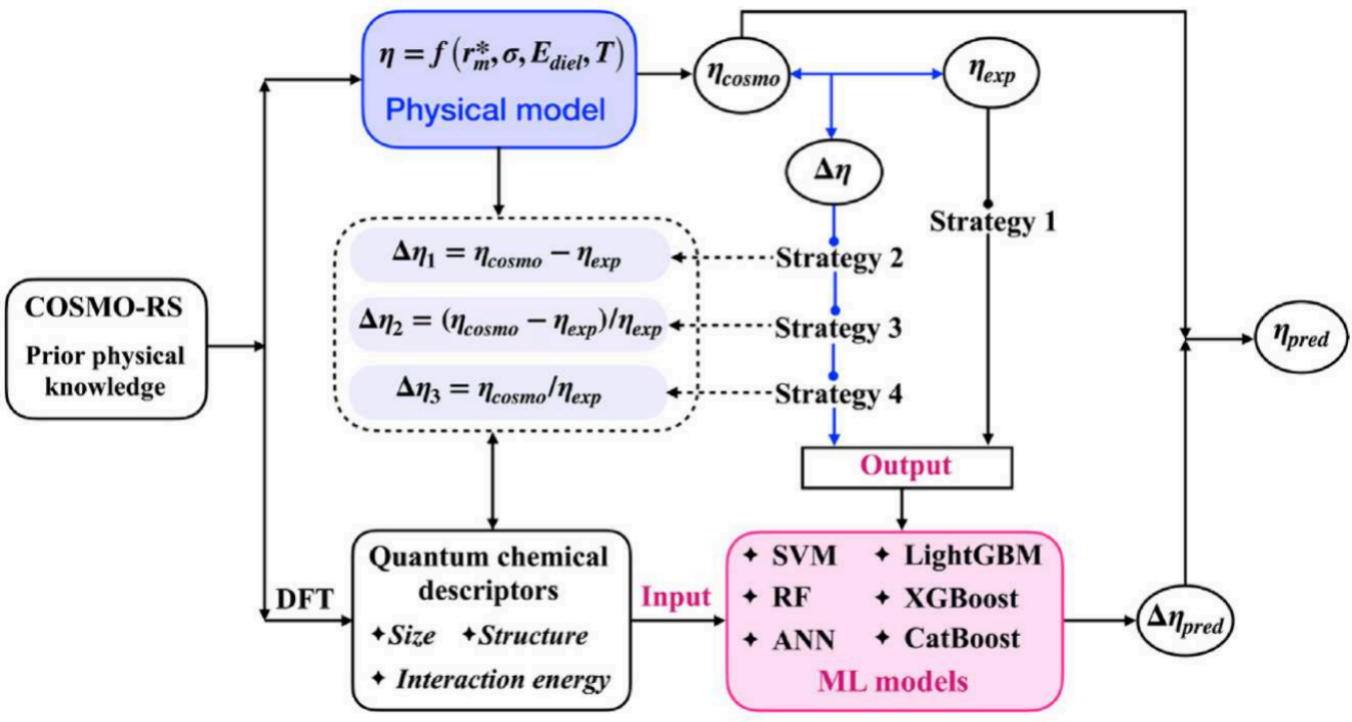
A schematic representation of the algorithm for training physics-based ANN algorithms for IL viscosity prediction. Reprinted from Fan et al.^50^ Copyright 2024 Institute of Process Engineering, Chinese Academy of Sciences under the CC BY-NC-ND license.

Fan et al.^50^ integrated physics-based COSMO-RS viscosity predictions into training ML models for IL viscosity prediction (Figure 3). By evaluating deviations between COSMO-RS predictions and experimental data, they enhanced model performance. For instance, an FFNN trained on relative discrepancies achieved *R*^2^ = 0.9934, compared to 0.8131 with experimental deviations alone, demonstrating the benefits of incorporating physical knowledge into complex property modeling.

Bavoh et al.^55^ used FNNs to predict methane hydrate phase boundary equilibrium temperatures in ILs. Evaluating various optimization algorithms and activation functions, they found “Adadelta“ with “elu” to be optimal (*R*^2^ = 0.981), highlighting the advantages of thorough hyperparameter optimization.

Emerging research also focuses on predicting IL toxicity using ANNs. Yan et al.^25^ created a curated database of 183 ILs with 6700 toxicity data points, achieving *R*^2^ values from (0.76 to 0.82). However, there is room for improvement. Hodyna et al.^24^ used QSAR modeling and an Associative Neural Network (ASNN) to predict IL cytotoxicity, achieving a cross-validated determination coefficient of 0.75. Despite satisfactory results, overfitting and data set limitations remain challenges.

Table 1 summarizes selected results from recent work on modeling IL properties using FNNs and CFNNs. It can be seen that, in most cases, ANNs give better results than conventional models, especially when large and diverse data sets are used. However, occasionally, the pursuit of the best possible results leads to the creation of overly complex models that give good results in theory, but their applicability outside the database used is severely limited due to overfitting. It should be noted that most of the models listed in Table 1 incorporate both structural and temperature/pressure dependencies simultaneously. Presenting combined modeling results without separately demonstrating the model’s ability to predict properties based solely on structure or solely on environmental conditions is potentially misleading and may result in inflated performance metrics. Importantly, the primary objective of these IL property prediction tasks is not merely to interpolate properties between different conditions (a task that can be achieved without complex ANNs and can be effectively modeled with simpler physical functions), but rather to predict the properties of novel, previously unseen structures. An illustrative example of disjointed modeling approaches, where structural dependencies are treated separately from working conditions (correction), can be found in the series of works by Paduszyński.^56−58^

In summary, while FFNNs and their variants are highly effective in modeling IL properties, factors like data set size, data splitting, and hyperparameter optimization significantly influence model performance.

### Convolutional Neural Networks

**Figure 4:**
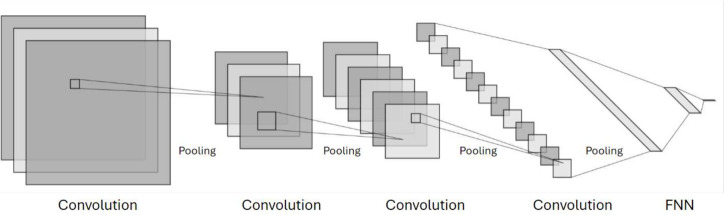
A scheme of a typical 2D-CNN using matrices as input. For 1D-CNNs, the matrices would be replaced by vectors.

Convolutional neural networks (CNNs) are deep neural networks specifically designed for visual data analysis. They employ convolutional layers to extract features and pooling layers to reduce dimensionality (Figure 4). CNNs excel in tasks such as image classification and object detection due to their ability to learn hierarchical representations from raw data. Input data for CNNs is typically organized into 3D matrices (2D-CNN); however, convolutions can also be applied to simple 2D vectors (1D-CNN). Hyperparameters to optimize during CNN training include the size and number of convolutional filters, the size and stride of the filter windows, the choice of activation function, and the depth and architecture of the network, which involves determining the number and arrangement of convolutional and pooling layers.

In property prediction, CNNs can be applied in two main ways: first, to calculate 2D feature vectors as inputs for other machine learning algorithms,^27,71^ such as FFANNs; and second, to directly predict specific parameters using preprocessed 2D or 3D input data sets.^26,72,73^

**Figure 5:**
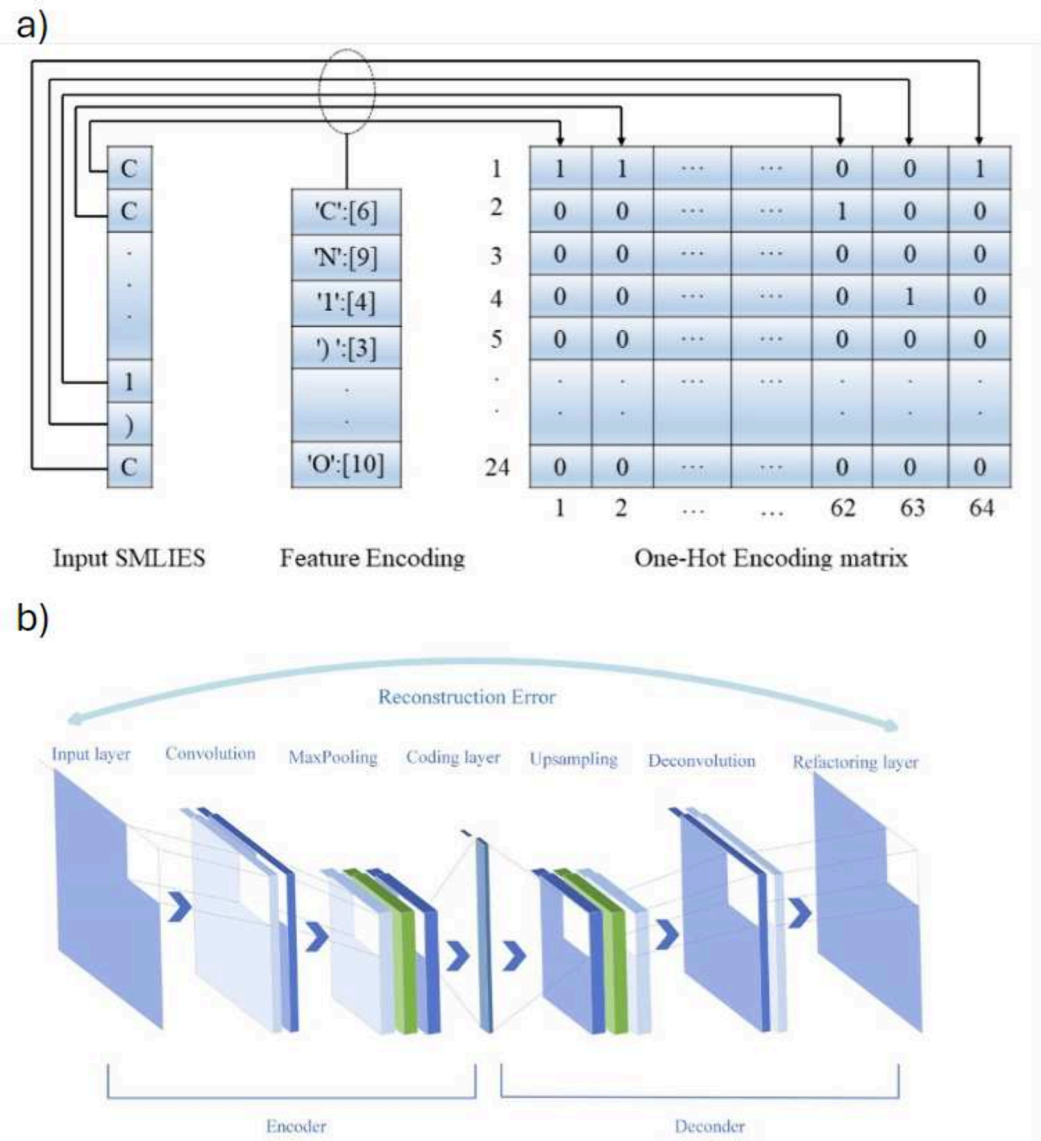
Structure encoding process from SMILES (a) and structure of the convolutional autoencoder (b). Reprinted with permission from Liu et al.^71^ Copyright 2023 John Wiley and Sons.

In the first approach, Liu et al.^71^ utilized matrices generated from one-hot encoded SMILES (Figure 5a) as input to a convolutional autoencoder (CAE) (Figure 5b) to reduce the dimensionality of the feature matrix to a vector (coding layer). A CAE is an unsupervised neural network that employs an Encoder-Bottleneck-Decoder architecture to create a latent space representation of the data set. This enables the network to learn representative patterns in the database and reconstruct them from a 2D vector. While CAEs are commonly used for image processing tasks like compression or anomaly detection, Liu applied them to compress SMILES structural encodings for subsequent use in MLP, RF, or SVM models. Their study showed that the compressed SMILES vectors effectively captured the complexity of ILs in a CO_2_ solubility prediction task, delivering results comparable to those of models based on RDKit-calculated features.

The second approach was adopted by Fan et al. in two contributions: one on predicting the toxicity of ILs against the leukemia rat cell line^26^ and another on modeling the infinite dilution activity coefficients of ionic liquid-solute systems.^72^ In the former study, an optimized 1D convolutional neural network (1D-CNN) was used to predict toxicity from a database of 155 ILs characterized by molecular fingerprints computed with RDKit and sigma profiles calculated using COSMO-SAC. The model achieved an *R*^2^ value of 0.965, indicating reliable predictions, though a lack of direct comparisons with other models limits the assessment of its superiority. In the second study, they modeled infinite dilution activity coefficients using a database containing 52 ILs and 114 organic solutes. Comparisons of a two-layer FNNN, CNN, and CDNN (a CNN combined with a two-layer FFNN for output calculation) using RDKit descriptors revealed that the CDNN outperformed both other neural networks and algorithms such as boosting, RF, and SVM.

In a related study, Esmaeili et al.^73^ investigated the prediction of the refractive index using six ML techniques, including 1D-CNN and boosting algorithms. Among models trained on a data set of 6098 data points characterized by temperature, wavelength, and chemical substructure encoded using the GC method, XGBoost performed best. However, the lack of optimization for CNN architecture and hyperparameters limits the study′s implications.

Makarov et al.^12^ used a transformer-CNN^74^ to predict the melting temperature of ILs using 2212 data points. The transformer-CNN, trained via the OCHEM tool,^23^ leverages a transformer architecture^75^ with an attention mechanism to calculate latent representations of input SMILES (it is noteworthy that transformers are currently a leading architecture in natural language processing applications, including large language models like ChatGPT and translation tools). Makarov′s model, pretrained on 1.7 million SMILES, provided input to a 1D-CNN architecture. Compared with RF models trained on various molecular descriptors, the attention-based Transformer-CNN achieved superior results *R*^2^ = 0.67 and prediction root mean squared error (RMSE) of 44 °C.

### Recurrent Neural Networks

Recurrent neural networks (RNNs) are a neural network architecture particularly suited for processing sequential data due to their ability to capture and utilize information from previous time steps. Unlike FFANNs, RNNs feature recurrent connections that create directed cycles, enabling dynamic temporal behavior. Input data for RNNs typically consists of sequential data points, each potentially comprising multiple features. For example, in natural language processing tasks, data points may correspond to words or characters, with features represented as word embeddings or one-hot encoded vectors. Optimizing RNN performance involves tuning several hyperparameters, including the number of recurrent units (or cells), the learning rate, the type of RNN cell architecture (e.g., basic RNN, long short-term memory (LSTM), or gated recurrent unit (GRU)), and the sequence length, which determines the extent of the input sequence during training.

In the context of IL property prediction, RNNs are not commonly used due to their inherent structural limitations, as evidenced by limited adoption in the reviewed literature. Mousavi et al. employed an RNN framework to predict H_2_S solubility in ILs, comparing its performance with other machine learning methods, including CNNs, FNNs, DBNs, and deep neural networks initialized with decision trees. Their RNN architecture incorporated GRUs and LSTM cells to address challenges like vanishing and exploding gradients. The models were trained on a data set comprising 1516 data points corresponding to *H*_2_*S* solubility across 37 diverse ILs, with chemical descriptors calculated using the GC method. However, RNNs underperformed compared to other methods, with CNNs achieving the best results (*R*^2^ = 0.9989) while RNNs reached an *R*^2^ value of 0.9910.

**Figure 6:**
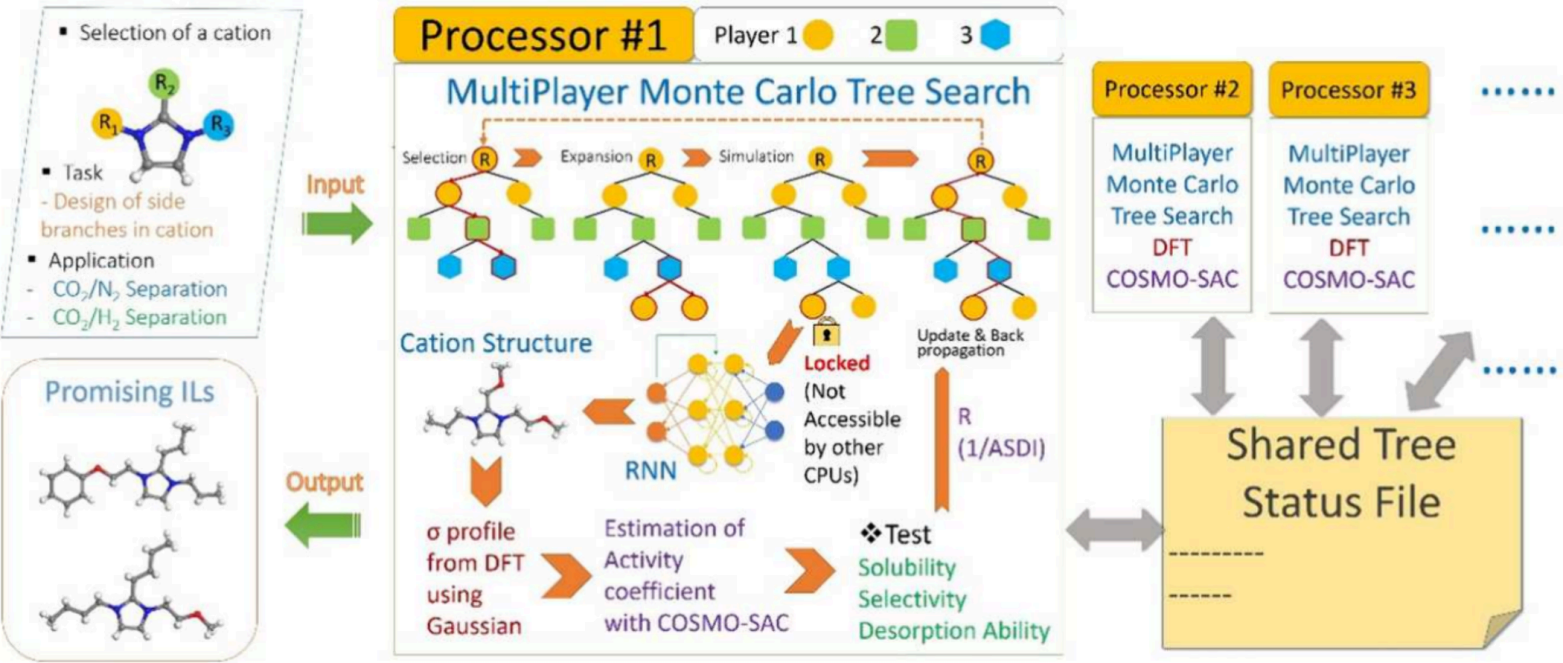
A schematic of the algorithm for designing application-specific ionic liquids. Reprinted with permission from Zhang et al.^76^ Copyright 2021 Elsevier.

In another study, Zhang et al.^76^ combined RNNs with multiplayer Monte Carlo Tree Search (MP-MCTS) to develop a parallel framework for generating and evaluating multiple ILs aimed at optimizing cation structures. A scheme of the algorithm is shown in Figure 6. The model was designed to produce cations with improved efficiency for carbon capture, with performance validated through activity coefficients calculated using COSMO-SAC. The RNN was trained on 414 972 SMILES strings from the MOSES database and used to determine the conditional probability distribution for the next symbol in a string. The results demonstrated the potential for designing high-performance ILs with targeted properties.

Davoudi and Ameri^77^ proposed a model for predicting water activity in ionic liquid-based ternary systems. Their study compared ten machine learning methods, including ANN approaches such as FNN, CFNN, RBF, and RNN, as well as conventional techniques like SVM, DT, and Gaussian Process Regression. Among all tested methods, ANN approaches achieved the best results, with CFNN outperforming the others.

### Graph Neural Networks

Graph neural networks (GNNs) are a powerful deep learning architecture specifically designed to analyze and process data structured as graphs. Unlike traditional neural networks, which primarily handle Euclidean data (e.g., vectors and matrices), GNNs excel at capturing the intrinsic relationships between entities within a graph. In molecular contexts, nodes in the graph represent atoms, while edges correspond to the chemical bonds connecting them. This enables GNNs to effectively learn from the intricate structures of molecules and their subcomponents. By iteratively aggregating and processing information from neighboring nodes and edges, GNNs build a comprehensive understanding of the entire molecule and its properties.^78,79^ Compared to conventional methods, which may struggle with capturing long-range interactions between distant atoms, GNNs excel in learning these crucial relationships within molecular graphs. This unique capability makes GNNs valuable for tasks such as predicting physicochemical properties relevant to material design. Although GNNs have demonstrated performance comparable to or superior to many conventional machine learning methods in various predictive tasks,^40^ there remains significant potential for improvement through architecture optimization and parameter tuning. Critical hyperparameters for GNNs include the number of layers, which affects the depth of the network and the range of node interactions it can capture, and the number of hidden units in each layer, which influences the model’s capacity. The choice of an information propagation algorithm, which governs how information from neighboring nodes is aggregated, also plays a crucial role in GNN effectiveness.^80^ Advanced graph pooling layers, which prevent information loss during aggregation, can further enhance GNN architectures.^81^

**Figure 7:**
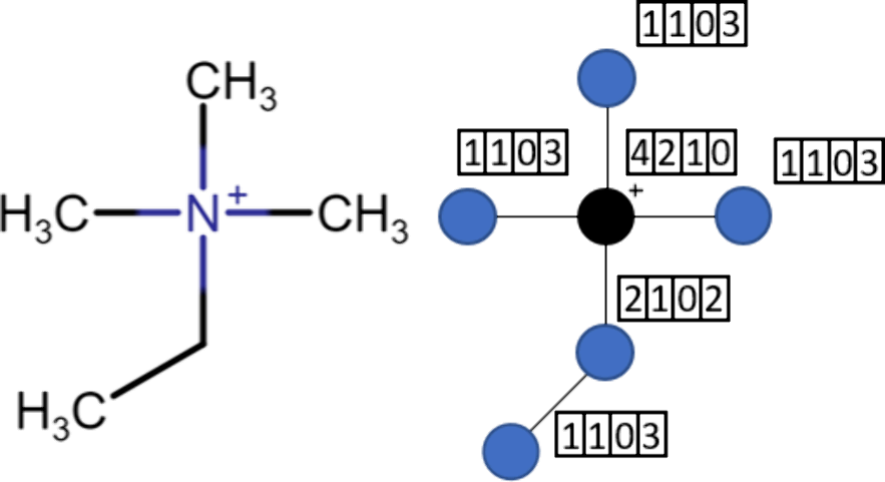
An ethyl(trimethyl) ammonium ion is represented as a graph, where additional parameters are assigned to each node to describe specific atomic properties (e.g., number of bonds, atom type, formal charge, number of bonded hydrogen atoms). Hydrogen atoms are omitted for simplicity.

Formally, a graph in GNNs is defined as , where  is a set of *N* nodes (atoms), and  is the set of *M* edges (bonds). An edge  connects nodes . If the graph includes node attributes (e.g., atomic mass, formal charge) or edge attributes (e.g., bond type, order), these are represented by a node attribute matrix  and an edge attribute matrix , where *D* is the number of atom attributes and *K* is the number of bond attributes. A simplified example of encoding an ion’s structure into a GNN-friendly format is shown in Figure 7. In ILs, graph representation is more complex due to the presence of two distinct molecules (cations and anions). These can be represented as separate graphs, which are connected only after the final readout, or within a single graph, either connected or disconnected by an ionic bond. Recent research suggests that the choice of representation has minimal impact on model performance.^43^

In the field of IL property prediction, the application of GNNs remains relatively unexplored. Rittig et al.^79^ investigated the use of GNNs for predicting the temperature-dependent activity coefficients of ILs as solutes. Their model was compared to the matrix completion method (MCM) introduced by Chen et al.^82^ After extensive optimization and training on a data set of 41 553 experimental data points, the GNN model achieved results comparable to MCM while demonstrating superior generalizability, accurately predicting the activity coefficients of IL solutions containing molecules not present in the training data.

In a recent study, Jian et al.^84^ applied GNNs to predict CO_2_ absorption in ILs. They compared GNN models with traditional molecular fingerprint (FP) and GC encoding methods using machine learning algorithms such as SVM, RF, XGBoost, and MLP. During optimization, they tested three information propagation algorithms: graph convolution network (GCN),^85^ graph attention network (GAT),^86^ and graph isomorphism network (GIN).^87^ Their results indicated that the GIN algorithm provided the best performance, achieving an *R*^2^ value of 0.9884. Similar performance was observed with GAT and the MLP/XGBoost models, irrespective of the chemical structure encoding method (FP or GC). Furthermore, Jian et al. used the GNN explainer method to better understand the model’s predictions. The explainer employs a learnable mask matrix applied to the graph’s adjacency matrix, identifying the most informative subgraphs through gradient-based optimization. Their findings highlighted that molecular fragments involved in chemical interactions with CO_2_ are generally more significant than those participating in physical interactions, aligning with the stronger nature of chemical bonding. The study also revealed that amine groups with varying hydrogen counts have different levels of importance in the CO_2_ absorption process.

Baran and Kłoskowski have recently carried out a comprehensive study on the use of GNNs for predicting common IL properties, including surface tension, viscosity, and density.^43^ Their analysis covered aspects such as outlier detection and data cleaning, data splitting strategies, electrostatic information, structure-to-graph conversion methods, and information propagation algorithms. They also explored transfer learning and fine-tuning techniques to enhance GNN performance. Their key findings indicated that GNN models for density and surface tension performed optimally with raw data sets, while the viscosity model achieved the best results with a smaller, cleaned data set. As noted previously, this suggests that more diverse data sets, even with some mislabeled entries, may benefit neural network applications. Median absolute deviation outlier detection was crucial for achieving optimal performance in the density model. Additionally, the choice of a convolution function, such as GCN, GAT, or molecular fingerprint-based networks,^88^ significantly impacted performance, emphasizing the importance of hyperparameter optimization. Transfer learning was also shown to be beneficial, with patterns learned from the best-performing density model improving the performance of viscosity and surface tension models through fine-tuning.

**Figure 8:**
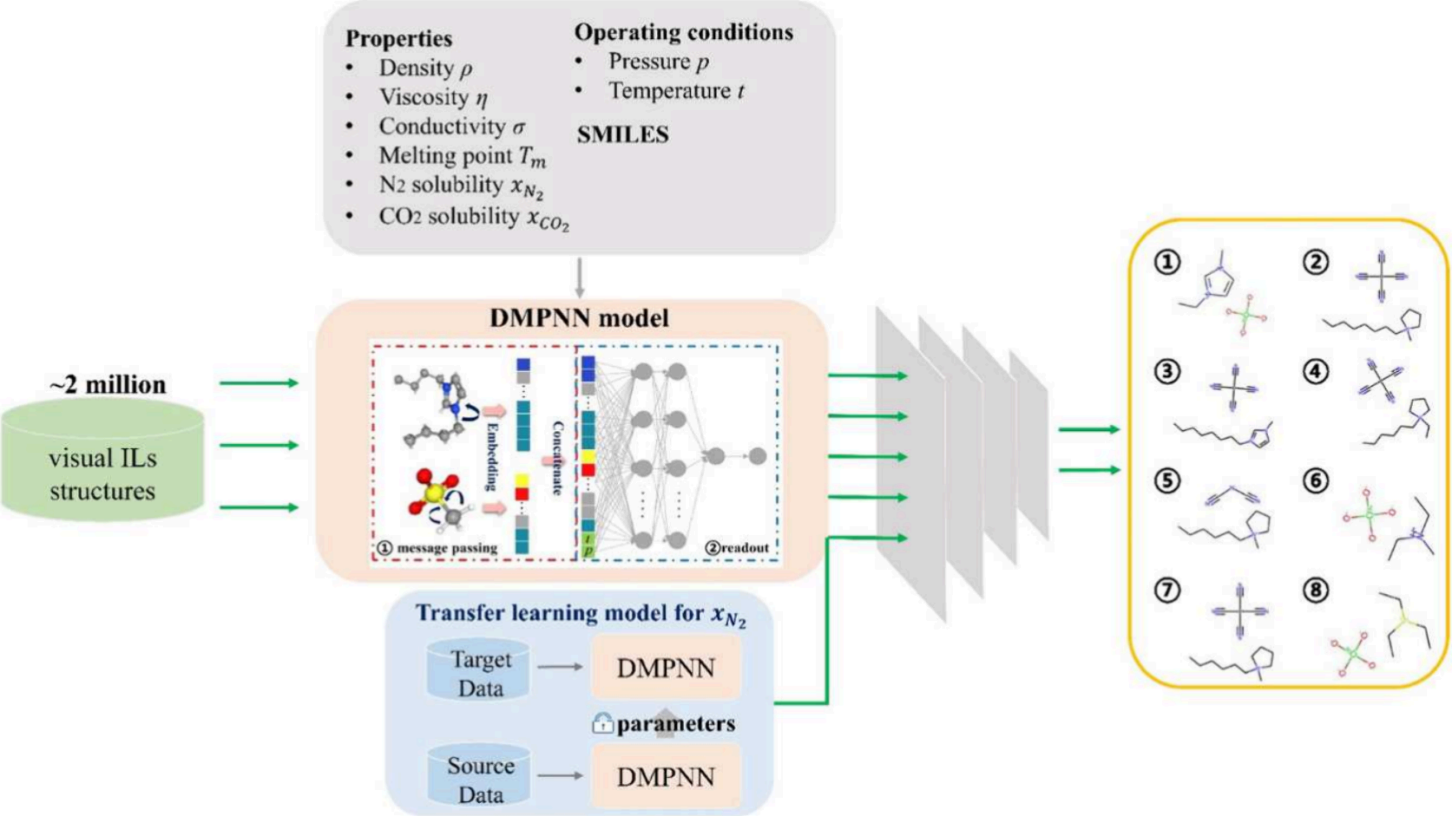
An overview of the training process for a GNN model and electrolyte screening for NRR and CO2RR reactions. Reprinted with permission from Song et al.^83^ Copyright 2023 Elsevier.

Another group applied GNNs to predict six IL properties (viscosity, conductivity, melting point, density, N_2_ solubility, and CO_2_ solubility) to identify promising ion combinations for electrolytes in nitrogen reduction and electrocatalytic carbon dioxide reduction reactions,^83^ as illustrated in Figure 8. The directed message-passing neural network (DMPNN) outperformed traditional machine learning models like SVM and FFNN, which relied on molecular fingerprints. For smaller data sets, such as nitrogen solubility, transfer learning improved GNN predictions. Through high-throughput screening, the study identified eight synthesizable ILs that outperformed the reference [P_14,6,6,6_][eFAP].

## Evaluation and Interpretability

The application of ANNs to the modeling of ILs has demonstrated significant advancements, owing to their ability to capture complex relationships and predict various physicochemical properties with high accuracy. This section evaluates and interprets these ANN models, focusing on the methodologies used to assess their performance, the metrics applied to quantify predictive capabilities, and the strategies employed to understand the mechanisms driving their predictions. By critically analyzing these aspects, this review aims to provide a comprehensive understanding of the current state of ANN-based modeling for ILs and to identify key areas for future improvement. This evaluation not only benchmarks the effectiveness of different ANN architectures and training protocols but also highlights the interpretability of the models, which is essential for gaining scientific insights and ensuring their practical applicability in IL research.

**Figure 9:**
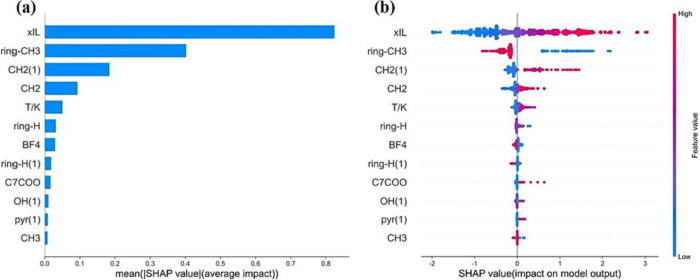
SHAP summary plot (a), SHAP feature importance plot (b), and SHAP interaction plot. Reprinted with permission from Liu et al.^68^ Copyright 2023 Elsevier.

Understanding why a model makes a specific prediction is often as important as the prediction’s accuracy, particularly in applications requiring interpretability. Several methods are available to provide such insights, with SHAP (SHapley Additive exPlanations) emerging as a popular technique. SHAP, a Python-based “model interpretation” package, can interpret the output of any machine learning model.^89^ For a given prediction, it assigns an importance value to each feature. Liu et al.^68^ employed SHAP to evaluate the significance of various factors—such as the molar fraction of IL, pressure, temperature, and structural fragments—in predicting heat capacity and density for IL binary mixtures with organic solvents. Figure 9 presents key plots from their SHAP analysis. Figure 9a illustrates the average impact of each feature on heat capacity predictions using an ANN model, showing that the molar fraction of IL has the greatest influence, followed by the ring CH_3_ and methylene substituent. Figure 9b demonstrates whether specific features positively or negatively affect heat capacity, with feature values represented by a gradient from blue to red. The results indicate that high IL molar fractions and a substantial presence of CH_2_ groups positively influence heat capacity in binary mixtures.

Chen et al.^90^ constructed predictive models for viscosity, density, heat capacity, and surface tension of IL-IL binary mixtures using the GC method, which is highly compatible with SHAP interpretation. Their analysis revealed that temperature and the mole fraction of ILs are the most critical factors for predicting viscosity and heat capacity, as expected. Additionally, chemical structure significantly influenced density, with [BF_4_] and CH_2_ groups identified as the predominant contributors.

Mohan et al.^91^ compared the performance of different machine learning architectures for predicting the temperature- and pressure-dependent surface tension and speed of sound, using sigma profiles calculated via COSMO-RS as input features. Gradient boosting trees (GBT) and SVM achieved the best results for surface tension and speed of sound predictions, respectively. SHAP analysis was employed to compare the impact of features across models, including XGBoost, RF, FFNN, and GBT for surface tension predictions. Despite similar performance metrics across these models, tree-based models (GBT and RF) relied heavily on the molecular surface area, nonpolar sigma profile regions (S5 and S9), and temperature for predictions. In contrast, the FFNN model emphasized anion polar sigma profile features (S11–S13) and IL molecular weight. Lower surface area, sigma profile regions, and temperature increased surface tension, consistent with experimental findings showing that surface tension depends on nonpolar moiety size (e.g., alkyl chain length). Longer alkyl chains reduce surface tension due to weaker Coulombic interactions. However, SHAP analysis revealed that the FFNN model predicted higher surface tension with lower anion polarity (S11–S13), contradicting experimental measurements and likely contributing to the model’s poorer performance.

## Other Related Work

In this section, we highlight notable studies that extend beyond traditional IL property prediction, emphasizing the potential of computational methods to address challenges from various perspectives, thereby saving significant time and resources. ANNs play a critical role in this effort, offering efficiency and simplicity while combating the “black box” stereotype through interpretative techniques.

Replacing time-consuming and costly molecular dynamics simulations with machine learning methods presents significant promise for dimensionality reduction and learning the potential of mean force. Ruza et al.^92^ introduced a dual GNN architecture to model the potential of mean force for coarse-grained simulations of ILs, effectively separating inter- and intramolecular interactions. By incorporating temperature as an input, the model achieves temperature transferability. Validated against all-atom simulation data, the GNN model accurately reproduces structural and dynamic properties across varying temperatures, advancing the coarse-graining of complex systems with enhanced efficiency and reduced computational costs.

The future of IL research lies in designing new cations and anions to create tailored combinations with desired properties. Integrating this process with artificial intelligence models capable of planning synthesis pathways^93^ could lead to the development of innovative materials with minimal experimental effort. Liu et al.^94^ proposed a novel framework for designing ILs for carbon capture applications, combining syntax-directed variational autoencoder (SDVAE),^95^ DeepFM,^96^ and gradient-aided particle swarm optimization (GBPSO). SDVAE ensures the generation of chemically valid SMILES strings by adhering to syntactic rules, DeepFM predicts IL solubility using factorization machines and MLP, and GBPSO optimizes latent space coordinates for SDVAE to generate ILs with promising CO_2_ capture properties. This synergistic approach enabled the design and experimental validation of a novel IL with excellent carbon capture potential.

Mian et al.^97^ utilized an FFANN optimized with the gray-wolf optimization (GWO) algorithm to develop a predictive model for determining process parameters in the preparation of IL-based gas separation membranes. This approach reduced the number of experimental trials required for optimal membrane design. Their analysis compared the performance of an FFNN optimized with gradient descent against one optimized with GWO. The original back-propagation neural network (BPNN) model achieved 95.22% accuracy, while the GWO-optimized BPNN achieved a higher accuracy of 97.98%.

Another research group applied ML methods to predict the yield of CO_2_ cyclization reactions catalyzed by ILs.^29^ They initially compiled a data set of 866 complete experimental data points, which included details on catalyst structures, reactants, products, experimental conditions, and reaction yields. Molecular descriptors calculated using density functional theory (DFT) augmented the data set. They then trained RF, SVM, and MLP models to predict reaction yields. However, selecting an appropriate set of input features proved challenging, leading to large discrepancies between training and testing metrics. The researchers hypothesized that solvents or additives might significantly impact reaction yield. Due to the diversity of these variables, finding a suitable descriptor was difficult, particularly because IL catalysts may also serve as solvents. As a solution, they replaced solvent/additive information with binary descriptors to distinguish between reactions with and without solvents or additives, or excluded this information entirely. This approach improved model performance but compromised its generalization capability.

## Conclusion and Future Perspectives

The application of ANNs in modeling ILs has significantly advanced our ability to predict their thermodynamic and physical properties. The integration of various types of ANNs has often demonstrated superior predictive capabilities compared to traditional models. CNNs, in particular, excel at efficient feature extraction, often outperforming FFNNs in predictive accuracy. Meanwhile, the growing popularity of GNNs lies in their straightforward handling of graph-based molecular representations, enabling the rapid prediction of parameters even for compounds not included in the training data set. These advances highlight the critical importance of robust data preparation, including meticulous data collection, feature engineering, and data cleaning, in developing accurate and reliable models.

Despite these advancements, challenges persist, particularly regarding model complexity and the risk of overfitting. Overly complex models, while theoretically capable of high accuracy, often fail to generalize beyond their training data sets. Additionally, such models demand significantly more computational resources, increasing training time and energy consumption without necessarily delivering proportional improvements in performance. Future research should prioritize enhancing the generalizability and efficiency of these models by employing techniques such as regularization, ensemble methods, and rigorous cross-validation strategies to mitigate overfitting.

Current methods for predicting the molecular properties of ILs lag behind SOTA approaches in molecular property prediction, which increasingly leverage advanced self-learning techniques, transformer-based architectures, and graph neural networks. While ANNs, including CNNs and GNNs, have improved IL property modeling, their application remains limited by data quality, generalization issues, and computational costs. In contrast, modern self-supervised models such as Mol-BERT, MolCLR, and XGraphBoost excel at extracting meaningful representations from large-scale unlabeled molecular data sets, enabling superior predictive performance. Bridging this gap by integrating IL modeling with these advanced techniques could significantly enhance accuracy, efficiency, and generalizability.

Furthermore, the integration of ANNs with other computational methods presents a promising avenue for designing novel ILs with targeted properties. Combining ANNs with optimization algorithms and employing advanced interpretative tools like SHAP can offer deeper insights into feature importance and model behavior. These integrated approaches have the potential to significantly reduce the time and resources required for experimental validation, enabling rapid innovation in IL development and applications.

In conclusion, while significant strides have been made in using ANNs for IL modeling, continued research and innovation are essential. By addressing current challenges and leveraging emerging computational techniques, we can unlock the full potential of ANNs to design ILs with tailored properties, paving the way for transformative advancements in industrial applications.
